# The development of a clinical research educational training for community health workers using the joint task force for clinical trial competency framework

**DOI:** 10.3389/fphar.2023.1295281

**Published:** 2023-12-07

**Authors:** Amin Yakubov, Dina Pimenova, Alzahraa Ahmed, Romelia Corvacho, Joanna Madigan, Jay Naik, Chen Lyu, Anita McFarlane, Victoria Foster, Megan Haseltine, Alexandr Trifonov, Ivette Cabrera, Clarissa Rios, Rachel Gross, Melanie Jay, Aaron Lord, Gabrielle Gold-von Simson, Brita Roy, Amy Freeman, Nadia Islam, James Holahan

**Affiliations:** ^1^ Langone Medical Center, New York University, New York, NY, United States; ^2^ NYU Clinical and Translational Science Institute, New York, NY, United States

**Keywords:** community health worker (CHW), community centered research, clinical research education, workforce development, diversity in research, clinical research

## Abstract

**Introduction:** The NYU Clinical & Translational Science Institute, in collaboration with a number of community-engaged initiatives, developed a training for community health workers (CHWs) to enhance health literacy about clinical research. This innovative research training provides CHWs with a basic level of competency in clinical research to convey the importance of research to communities and better advocate for their health needs. CHWs are an underutilized resource to engage diverse populations in clinical research. The training also addresses the need to expand and diversify the clinical research workforce—integrating CHWs into research teams and connecting underserved populations with research opportunities to enhance quality of care.

**Methods:** Structured individual interviews and focus group sessions were held with CHWs as well as clinical research faculty and staff to identify knowledge gaps in clinical research and identify best practices for educating community members on research. Using the Joint Task Force (JTF) for Clinical Trial Competency framework, an online course was developed consisting of 28 modules offered asynchronously for internal and external audiences. Topics include the fundamentals of clinical research, scientific concepts and research design, research ethics, study management, clinical study operations, communications, and teamwork, as well as the importance of diversity and equity in research and the barriers to participation.

**Results:** Learning was evaluated using multiple choice questions after each module to ensure the fundamental level of knowledge was obtained. A separate survey, completed at the conclusion of the course, evaluated the quality of training.

**Discussion:** The course aims to enhance the knowledge and skills of CHWs to help promote greater understanding of clinical research within the communities they serve, including the risks and benefits of clinical research and opportunities for participation. As members of the research team, community stakeholders can help design interventions tailored to the unique needs, culture, and context of their communities. In addition, this research training equips trainees with skills to engage the community actively, involving them in the research process and ensuring community priorities are represented in research through more community engaged processes.

## Introduction

Community Health Worker (CHW) is an umbrella term for an array of health practitioners who operate under various titles globally and whose overarching mission is to serve and engage the needs of culturally distinct communities toward improving health outcomes. Titles include: CHW, patient navigator, promotora, outreach specialist, community advocate, and community health educator, among others (CACHW.org, 2023). CHWs are essential frontline public health professionals who leverage their intimate understanding of local communities and often serve as trusted intermediaries between the members of those communities and both medical and social service systems. Equipped with an understanding of their community’s cultural characteristics, behaviors, and attitudes, CHWs are uniquely positioned to explain and navigate individuals through complex health systems and to communicate individual, family, and community-level needs to service providers to improve access to, and quality of, care. Through this integral “bridging” work, CHWs enhance the self-sufficiency and knowledge of community members and the community itself, strengthen relationships with service delivery agencies, and influence attitudes and practices through education, informal counseling, social support, and advocacy (Jackson and Gracia, 2014; Olaniran et al., 2017; American Public Health Association, 2019).

The key roles CHWs fulfill within the health service delivery landscape is demonstrated by their increased recognition within federal health-related legislation and strategic planning. In 2009, the US Bureau of Labor Statistics identified CHW as a Standard Occupational Classification, and the Department of Health and Human Services included CHWs within its five overall goals for reducing health disparities (Koh et al., 2011; Malcarney et al., 2017). The Patient Protection and Affordable Care Act (Public Law 111–148) and Health Care and Education Reconciliation Act of 2010 (Public Law 111–152) further encourages CHW integration into healthcare settings (Public Law 111-148 111th Congress Act, 2010; Public Law 111-152 111th Congress Act, 2010; Islam et al., 2015; Rodriguez, 2022). The Centers for Disease Control and Prevention funds CHW projects in multiple therapeutic areas, including heart disease, diabetes, and COVID-19 and in 2022 S.3479—Building a Sustainable Workforce for Healthy Communities Act was introduced in the Senate, a bill reauthorizing and revising a CDC grant program to develop and/or expand CHW programs (Congress, 2021; Rodriguez, 2022). Despite an increase in national recognition, CHWs still lack standardized certification and training requirements that are consistent across states. The trainings often focus on CHW core competencies such as communication, individual and community assessment, and outreach skills, but do not include information on clinical research (Rosenthal et al., 2014-2022). For example, at NYU Langone Health (NYULH), the CHW training programs focus on various health areas, including Alzheimer’s Disease, behavioral health, diabetes, epilepsy, substance use, cancer, HIV, heart disease, hypertension, and social determinants of health (SDOH) (e.g., housing, food security, and federal benefits). Job preparedness for these programs is provided via CHW core competency training developed and promoted by community colleges and community-based organizations (CBOs), which is supplemented by project-specific training unique to the role of the CHW.

CHWs focus primarily on service delivery and health promotion but their contribution to research spans recruitment, outreach, survey implementation and administration, focus group facilitation, SDOH support, and disseminating data and results to communities in ways tailored to them. Despite “Evaluation and Research Skills” being a recognized CHW Core Competency by Rosenthal et al. (Rosenthal et al., 2014-2022), many CHWs lack training in the fundamentals of research, including scientific concepts, study design and methodology, biomedical ethics, and barriers to recruitment and retention of research participants. It is particularly important for CHWs who work with racial and ethnic minorities and immigrant populations to be knowledgeable of these barriers, which include logistical concerns, lack of insurance coverage, and historical mistrust of research and the healthcare system due to past exploitation. Involving CHWs in the research process can be one way of overcoming these barriers (Killough et al., 2023). CHWs can provide social support, build trust, and act as intermediaries between underrepresented communities and researchers to ensure that study materials, such as recruitment methods, are actionable for community members. For example, CHWs can navigate complex healthcare systems, provide language support, engage in health education, and reduce barriers to care by addressing financial toxicities—such as commuting expenses, child and family care, unemployment, and food insecurity—through referrals, continuous follow-up, and coordination with the appropriate entities. As the CHW profession evolves, these health professionals will play a pivotal role in collecting and reporting information related to the health status of community members, which is imperative for research design, implementation, and recruitment (Olaniran et al., 2017). This development will also address calls to open new career paths for CHWs in various research fields and expand the CHW workforce (10; 14).

A well-trained community-based workforce in research is better prepared to engage the community actively while enhancing their knowledge of research and participation in trials. In addition, as we recruit more diverse participants into clinical research, it will be imperative to have a community-centered, multi-lingual, and diverse, research-trained workforce that reflects the population (Murphy et al., 2023). For example, CHWs who understand their clients’ health conditions are able to navigate them to eligible therapeutic clinical trials. By understanding the availability of research options, CHWs can lower barriers to recruitment including by dispelling myths and misconceptions about research and reducing social and economic barriers. A research-knowledgeable workforce of CHWs can engage simultaneously with the community and researchers to help promote, study, and address the needs of the community, ultimately helping improve the health of their clients and communities (Killough et al., 2023). This contributes to translational science by ensuring that innovations progress not only unidirectionally from the bench to the bedside and community, but also cyclically back from the community again (Plasencia et al., 2023). For example, CHWs can help inform researchers on the ongoing state of their communities, such as trends of cancer diagnoses, environmental exposures, or social needs impacting health, thus creating an ongoing feedback loop of the health and social needs of the community (Plasencia et al., 2023). Lastly, as the COVID-19 pandemic has demonstrated, research demand can outpace the supply of clinical research professionals at times of public health emergencies (Freel et al., 2023). Expanding the capacity of the research workforce is essential for preparing for future outbreaks/emergencies and spikes in diseases among the population. CHWs can be a research-ready, highly-skilled, and trained workforce that can inform and respond to emerging disasters within their communities.

## Methods

**FIGURE 1:**
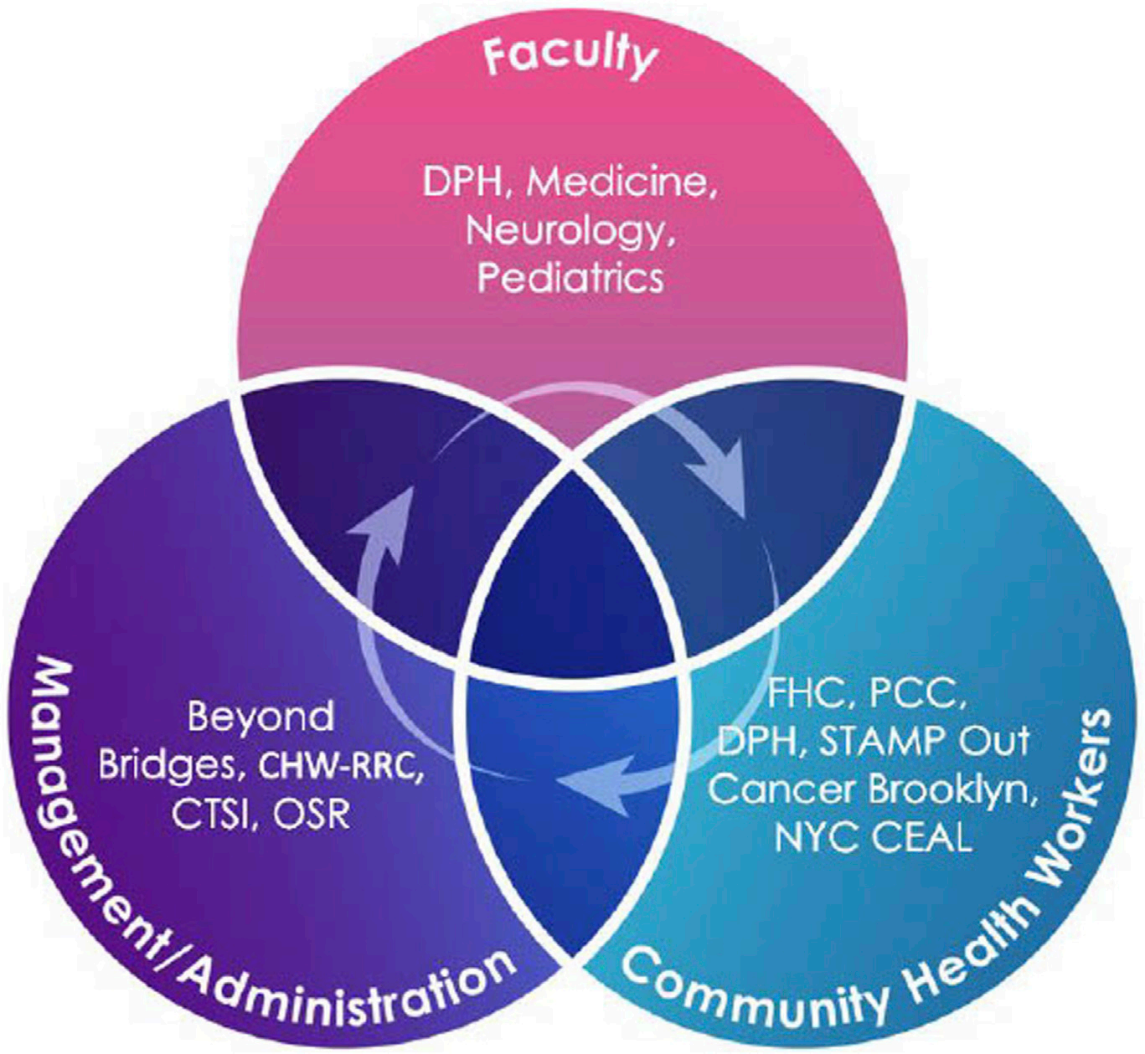
CHW Research Training Stakeholders. Stakeholders involved in the development of the training: the NYU Clinical & Translational Science Institute (CTSI), the NYU Community Health Worker Research & Resource Center (CHW-RRC), the Beyond Bridges Initiative, the Beatrice W. Welters Breast Health Outreach & Navigation Program, STAMP Out Cancer Brooklyn, the NY Community Engagement Alliance (NYCEAL) network, and departments of Neurology, Pediatrics, Medicine, and Population Health (DPH).

The Community Health Worker Research Training is an intra-institutional and multi-departmental initiative to train CHWs within the NYULH health system and nationwide. This initiative involved input and support from the NYU Clinical & Translational Science Institute (CTSI), the NYU Community Health Worker Research & Resource Center (CHW-RRC), the Beyond Bridges Initiative, the Beatrice W. Welters Breast Health Outreach & Navigation Program, STAMP Out Cancer Brooklyn, the New York Community Engagement Alliance (NYCEAL), NYU-CUNY Prevention Research Center (NYU-CUNY PRC), and the Office of Science and Research (OSR). Members of the CTSI Community Engagement and Population Health Research (CEPHR) group, along with NYU Grossman School of Medicine faculty and research staff, initially identified the need for a research training in response to a gap in culturally competent trainings for CHWs in the fundamentals of clinical research. Plasencia et al. recommend that CHW trainings are codeveloped with the participation of CHWs to leverage their in-depth knowledge of marginalized communities (Plasencia et al., 2023). This curriculum was codeveloped through a collaborative partnership with CHWs to ensure the training was informed by both research and community input. Feedback was obtained via individual interviews and group sessions with representatives from the Departments of Population Health, Medicine, Pediatrics, and Neurology, as well as the Perlmutter Cancer Center and the Family Health Centers (Figure 1). These feedback sessions with expert stakeholders identified several gaps in clinical research knowledge, including understanding research foundations and research processes, the drug and vaccine development process, identifying research opportunities, and experiences of research in minority and immigrant populations. Best practices were also identified by those with experience developing CHW training and education, who recommended that trainings be offered virtually, on-demand, and at no cost, and that they be easily accessible to all CHWs nationally.

The Joint Task Force (JTF) for Clinical Trial Competency was used as a framework for developing the curriculum, as it outlines a comprehensive set of competencies for clinical research professionals used by organizations worldwide (Sonstein and Jones, 2018). The JTF competencies include 47 leveled competency statements across eight domains that are expressed at a Basic, Skilled, and Advanced level. For the purpose of this training, we sought to convey a basic level of competency to promote a fundamental understanding of clinical research. Modules were selected based on knowledge gaps identified in interviewing faculty, staff, and CHWs. Examples of the competency-based training modules include scientific concepts and research design, ethical participation and safety considerations, development and regulation of investigational products, clinical study operations and good clinical practice, study and site management, data management and informatics, leadership and professionalism, and communications and teamwork. Given the unique role of CHWs, we included additional competencies to ensure that the experiences of research on Latine, Middle East and North Africa (MENA), Former Soviet Union (FSU), and East Asian populations were well represented. These include the history and ethics of biomedical and clinical research broadly and in particular regions such as Central and Latin America, MENA, FSU, and East Asia.

**FIGURE 2:**
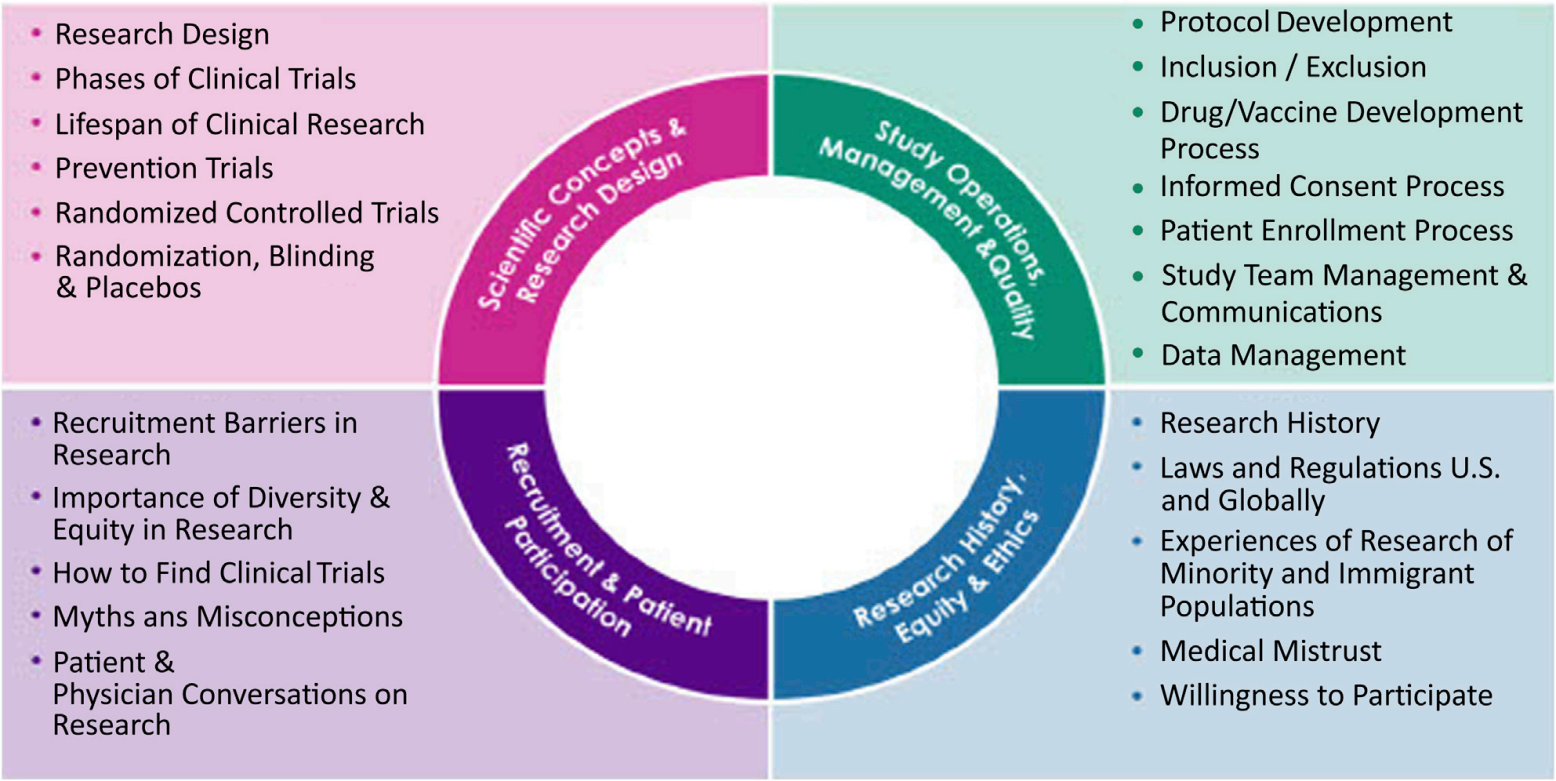
CHW Research Training Competencies. Research training competencies across 28 different modules on scientific concepts and research design, study operations, study management and quality, recruitment and patient participations, and research history, equity, and ethics. Seminar 1 includes the scientific concepts and research design, study operations, management and quality. Seminar 2 includes recruitment and patient participation and research history, equity and ethics.

By leveraging the JTF core competency framework, we created a research training that is tailored to CHWs and takes into consideration the populations we serve in New York City. The course includes 28 online, asynchronous modules grouped into 2 seminars. Seminar 1 (Foundations of Research) includes 20 modules while Seminar 2 (Research Ethics and the Importance of Diversity and Equity in Research) includes 8 modules (Figure 2). Each module is approximately 5 min long with voiceover narration provided by CHWs to represent a variety of voices and accents that reflect the intended target audience. The course is offered asynchronously for internal audiences via the NYULH FOCUS platform and external audiences via the RISE web-based platform. Trainees are required to complete multiple-choice quizzes after each module and answer all questions correctly in order to proceed to the next module, with the ability to retake quizzes as needed. Each module consisted of 1–4 questions assessing knowledge of the material presented. A separate exit survey was developed by research team members with experience in evaluation and survey design to gather feedback and evaluate perceptions of knowledge uptake, quality of training, and module preferences. This survey included multiple-choice, Likert scales, and free text responses. Open-ended text was coded into positive and negative responses and then further grouped into categories (e.g., applicability of training, relevance of content, quality of quiz questions). Participants who completed the exit survey were offered a $30 Amazon gift card for completion. The training was advertised to NYULH-affiliated CHWs through various mechanisms, including targeted e-mails and messages, tabling at events, and outreach to the CHW listservs. It was also advertised externally through partner organizations that engage CHWs, such as the CONNECT forum and Center for Community Health Alignment, and presented at the National Association of Community Health Workers (NACHW) Unity Conference in Austin, Texas (2023).

## Results

The participant responses were summarized using descriptive statistics in counts (%) for categorical variables. Chi-square test or Fisher’s exact test was used whenever appropriate to test the association between participants’ understanding of the basic concepts in Seminar 1 and Seminar 2 and individual module preferences. 428 participants accessed the training and of those, 318 completed training modules requiring a 100% score to proceed to the next module. 247 participants completed an optional post-training exit survey. Of those who completed the survey, 197 (80%) identified as CHWs, 32 (13%) identified as patient navigators, and 18 (7%) identified as other (community outreach coordinators, patient liaisons, navigators, social workers, research assistants, and managers). Trainees represented over 197 unique institutions nationwide, including academic medical centers, non-profit organizations, CHW networks, and private companies. When asked if they have completed prior research trainings, 79 (32%) respondents indicated that this was their first research training. Of those that have participated in research trainings in the past, 72 (29%) completed it in the past 12 months, 93 (38%) between 1–5 years ago, and 3 (1.0%) more than 5 years ago (Table 1).

**TABLE 1:** Post-exit survey participant responses.

Question	# (%)
Primary role	
CHW	197 (79.76)
Patient Navigator	32 (12.96)
Other	18 (7.29)
Last time you took a research training	
This was my first time	79 (31.98)
In the past 12 months	72 (29.15)
Between 1–5 years ago	93 (37.65)
More than 5 years ago	3 (1.21)
Scale question 1: How well do you understand the basic concepts of clinical research? Please rate on a scale of 0–5, with 0 being “not at all” and 5 being “very well”?	
0	6 (2.34)
1	7 (2.83)
2	19 (7.69)
3	67 (27.13)
4	78 (31.58)
5	70 (28.34)
Scale question 2: How well do you understand the history of clinical research? Please rate on a scale of 0–5, with 0 being “not at all” and 5 being “very well”?	
0	21 (8.5)
1	7 (2.83)
2	18 (7.29)
3	52 (21.05)
4	72 (29.15)
5	77 (31.17)
Scale question 3: How confident are you in applying the knowledge gained to your work? Please rate on a scale of 0–5, with 0 being “not at all confident” and 5 being “very confident?	
0	8 (3.24)
1	4 (1.62)
2	11 (4.45)
3	61 (24.70)
4	83 (33.60)
5	80 (32.39)
Recommend training to others	
Yes	247 (100)
No	0 (0)
Training provide practical skills	
Yes	245 (99.19)
No	2 (0.81)
Overall quality	
Poor	1 (0.4)
Average	116 (46.96)
Neutral	108 (43.72)
Good	22 (8.91)
Excellent	0 (0)
Top 5 most valuable modules (Seminar 1)	
Module 2 (Differences between clinical research and clinical trials)	151 (61.13)
Module 8 (What do we learn from clinical trials?)	126 (51.01)
Module 5 (Phases/lifespan of clinical trials)	124 (50.2)
Module 3 (Types of study designs)	120 (48.58)
Module 6 (The drug and vaccine development process step-by-step guide)	118 (47.77)
Top 5 most valuable modules (Seminar 2)	
Module 2 (Experiences of Research on Minority and Immigrant Populations, Medical Mistrust & Willingness to Participate (WTP) in Research)	159 (64.37)
Module 5 (Importance of diversity in clinical research)	146 (59.11)
Module 4 (Recruitment in clinical trials)	128 (51.82)
Module 3 (Why do clinical trials take so long? Trial enrollment process and barriers)	107 (43.32)
Module 1 (Barriers to research and historical events)	106 (42.91)

The participant responses summarized using descriptive statistics in counts (%) for categorical variables.

**FIGURE 3:**
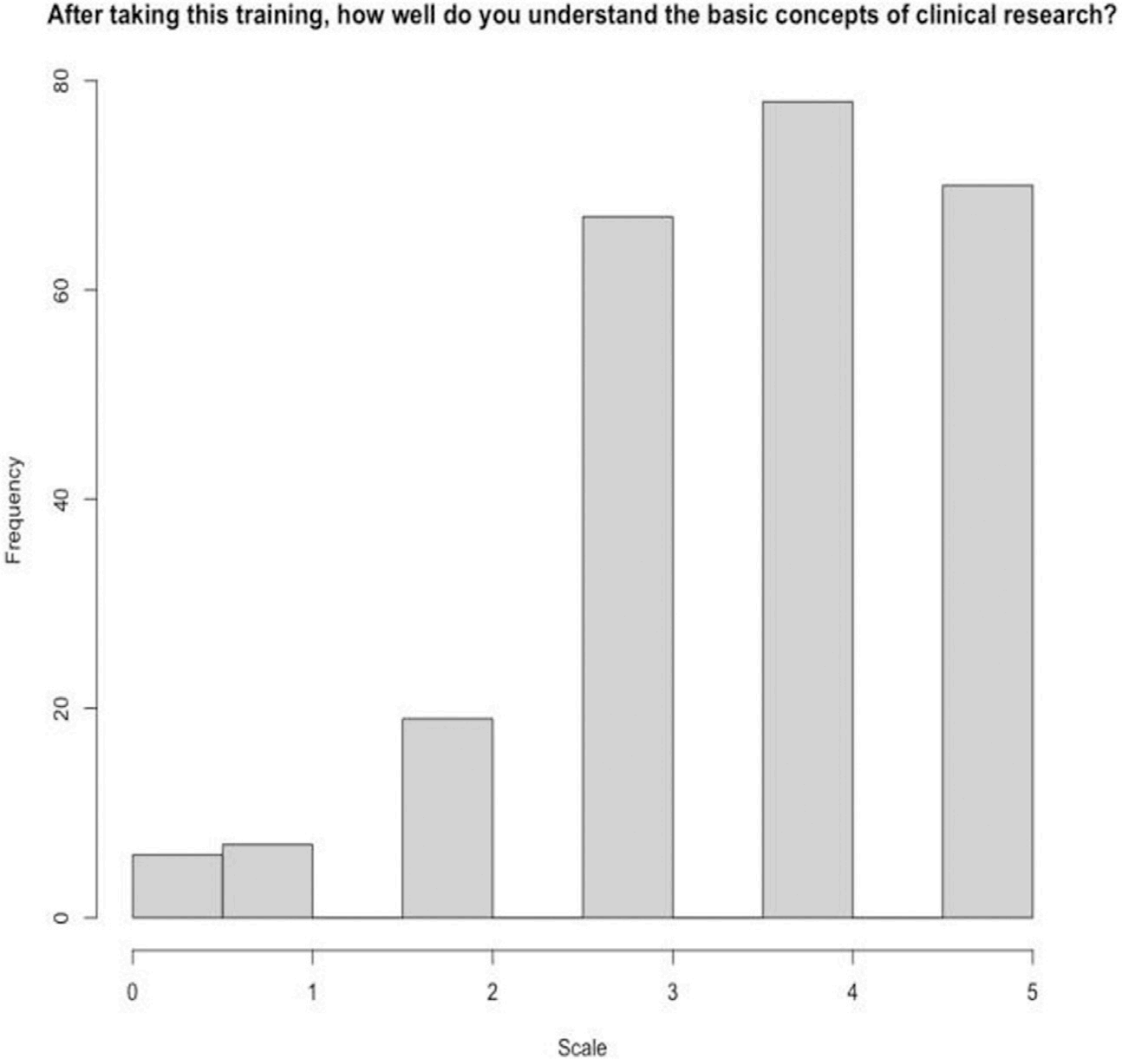
Understanding basic concepts of clinical research (seminar 1) (score 0–5).

**FIGURE 4:**
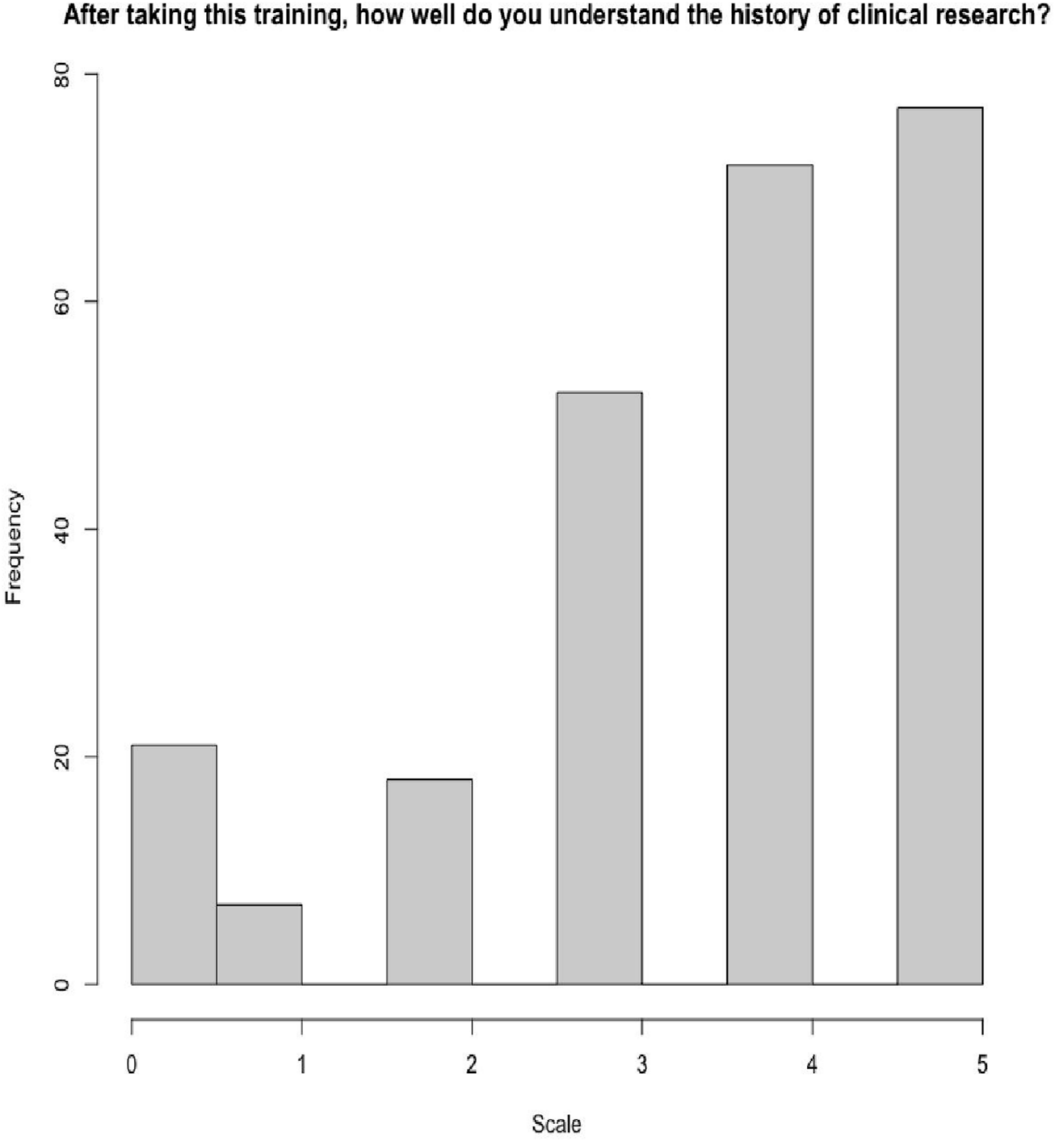
Understanding clinical research history and the importance of diversity and equity in research (seminar 2) (score 0–5).

As part of the exit survey, participants were asked to rate how well they understood the concepts presented in each of the seminars on a Likert scale from 0–5, with 0 meaning they did not understand the concepts at all and 5 meaning they understood the concepts very well. 148 (60%) of respondents indicated a 4 or higher on understanding the concepts in Seminar 1, which covered the foundations of clinical research, and 149 (60%) of respondents indicated a 4 or higher on understanding the concepts in Seminar 2, which covered the history of clinical research and the importance of diversity (Figures 3, 4). Participants were also asked to rate how confident they felt applying the training and knowledge to their everyday roles on a Likert scale from 0–5, 0 being not at all confident and 5 being very confident. 224 (91%) indicated a 3 or higher, indicating that they felt moderately confident to very confident. When asked if this training provided practical skills, 245 (99%) indicated favorably. 224 (91%) participants indicated that they found the quality of the training good or excellent and all 247 participants indicated that they would recommend this training to others (Table 1).

Participants were also asked to rate which modules they found most valuable to their overall learning and training experience. Seminar 1 (Foundations of Research) includes 20 modules while Seminar 2 (Research Ethics and the Importance of Diversity and Equity in Research) includes 8 modules (Table 2). The top 5 most valuable trainings in Seminar 1 included topics on the foundations of clinical research, the differences between study designs, protocol development, the drug and vaccine development process, and the importance of having power statistics in research. The top 5 most valuable trainings in Seminar 2 included topics on research history, understanding the influence of research on minority health, the lifespan of clinical trials, the importance of recruitment, and communicating with providers about clinical research. The most desirable trainings in both seminars included topics on the foundations of clinical research and clinical trials, and the understanding of the importance of diversity and equity in clinical research (Table 3).

**TABLE 2:** Seminars 1 and 2 module topics.

Seminar 1: Research foundations		
Module 0	Introduction to modules	
Module 1	Why clinical trials?	
Module 2	Differences between clinical research and clinical trials	
Module 3	Types of study designs	
Module 4	Phases of clinical trials	
Module 5	Phases/lifespan of clinical trials	
Module 6	The drug and vaccine development process step-by-step guide	
Module 7	What is a preventative trial?	
Module 8	What do we learn from clinical trials?	
Module 9	What is a protocol?	
Module 10	How are protocols designed?	
Module 11	What are protocol inclusion/exclusion criteria?	
Module 12	Importance of having number of participants and power statistics	
Module 13	Clinical trial terminology: What is randomization?	
Module 14	Randomized controlled trials	
Module 15	Clinical trial terminology: What is a placebo and blinding?	
Module 16	Clinical trial terminology: What is an informed consent?	
Module 17	Why participate in clinical trials?	
Module 18	Patient Enrollment Timeline	
Module 19	End of study	
Seminar 2: Research History and Importance of Diversity and Equity in Research		
Module 1	Barriers to research and historical events	
Module 2	Experiences of Research on Minority and Immigrant Populations, Medical Mistrust & Willingness to Participate (WTP) in Research	
Module 3	Why do clinical trials take so long? Trial enrollment process and barriers	
Module 4	Recruitment in clinical trials	
Module 5	Importance of diversity in clinical research	
Module 6	How to find and participate in clinical research	
Module 7	Myths & Misconceptions	
Module 8	Always ask your doctor about clinical research	

**TABLE 3:** Top 5 module preferences by role (seminar 1) and (seminar 2).

Module preferences (seminar 1)		CHW	Navigator	Other
Module 2	Checked	127	11	13
(Differences between clinical research and clinical trials)	Unchecked	70	7	19
Module 8	Checked	101	11	14
(What do we learn from clinical trials?)	Unchecked	96	7	18
Module 5	Checked	92	14	18
(Phases/lifespan of clinical trials)	Unchecked	105	4	14
Module 3	Checked	88	12	20
(Types of study designs)	Unchecked	109	6	12
Module 6	Checked	96	12	10
(The drug and vaccine development process step-by-step guide)	Unchecked	101	6	22
Module Preferences (Seminar 2)		CHW	Navigator	Other
Module 2	Checked	127	14	18
(Experiences of Research on Minority and Immigrant Populations, Medical Mistrust & Willingness to Participate (WTP) in Research)	Unchecked	70	4	14
Module 5	Checked	117	15	14
(Importance of diversity in clinical research)	Unchecked	80	3	18
Module 4	Checked	92	14	22
(Recruitment in clinical trials)	Unchecked	105	4	10
Module 3	Checked	85	10	12
(Why do clinical trials take so long? Trial enrollment process and barriers)	Unchecked	112	8	20
Module 1	Checked	79	13	14
(Barriers to research and historical events)	Unchecked	118	5	18

Top 5 most rated modules by role in seminar 1, including topics on the foundations of clinical research, the differences between study designs, protocol development, the drug and vaccine development process, and the importance of having power statistics in research. Top 5 most rated modules by role in seminar 2, including topics on research history, understanding the influence of research on minority health, the lifespan of clinical trials, the importance of recruitment, and communicating with providers about clinical research.

Lastly, 62 participants provided free text survey responses. Of those, 55 (84%) provided information on the importance of this course and how it fills gaps in knowledge, while 7 (11%) indicated that the quiz questions were too difficult. Free text responses included, “modules were so knowledgeable and helpful,” “as a [CHW] this course helped me a lot to understand the investigator process,” and “quizzes were difficult and tricky.” We also received personal messages from the majority of participants with feedback including, “I had a great time during this training session, even my colleague that was with me couldn’t help but join me,” “the training has revived my professional zeal,” and “the training was awesome and encouraging, this is the first time I feel that I am in the right profession.” When evaluating for understanding by seminar and module, we noted an association between top scored modules and a moderate understanding of the concepts. We noted that there was a significant relationship between the responses for scale question 1 and responses for each of the top most rated modules in seminar 1 (Table 4). Interestingly, of the top 5 rated modules in Seminar 1, modules 3, 5, and 8 were statistically significant in providing a score of 3 or higher in understanding basic concepts of clinical research. Similarly, we noted a statistically significant relationship for scale question 2 and responses for modules 1, 4, and 5 in Seminar 2. Of the top 5 rated modules in Seminar 2, modules 5, 6, and 8 were statistically significant in providing a score of 3 or higher in understanding the history of clinical research (Table 4). Furthermore, 224 (91%) participants indicated that they would apply the knowledge learned to their daily work and recommend the training to others (Table 1).

**TABLE 4:** Scale question 1 and 2. “After taking this training, how well do you understand the basic concepts of clinical research? Please rate on a scale of 0–5, with 0 being “not at all” and 5 being “very well”) by top 5 preferred modules (seminar 1). “After taking this training, how well do you understand the history of clinical research? Please rate on a scale of 0–5, with 0 being “not at all” and 5 being “very well”) by top 5 preferred modules (seminar 2).

Seminar 1 modules		Scale Q1 (foundations of clinical research by module preference)						
		0	1	2	3	4	5	*p*-value^a^
Module 2	Checked	6	7	9	48	36	45	<.001
(Differences between clinical research and clinical trials)	Unchecked	0	0	10	19	42	25	
Module 8	Checked	6	7	12	25	35	41	<.001
(What do we learn from clinical trials?)	Unchecked	0	0	7	42	43	29	
Module 5	Checked	0	0	7	28	39	50	<.001
(Phases/lifespan of clinical trials)	Unchecked	6	7	12	39	39	20	
Module 3	Checked	0	0	7	21	44	48	<.001
(Types of study designs)	Unchecked	6	7	12	46	34	22	
Module 6	Checked	6	7	4	34	26	41	<.001
(The drug and vaccine development process step-by-step guide)	Unchecked	0	0	15	33	52	29	
Seminar 2 Module		Scale Q2 (Research History by Module Preference)						
		0	1	2	3	4	5	*p*-value^a^
Module 2	Checked	10	5	13	37	48	46	0.42
(Experiences of Research on Minority and Immigrant Populations, Medical Mistrust & Willingness to Participate (WTP) in Research)	Unchecked	11	2	5	15	24	31	
Module 5	Checked	21	6	8	31	39	41	0.001
(Importance of diversity in clinical research)	Unchecked	0	1	10	21	33	36	
Module 4	Checked	0	1	3	30	39	55	<0.001
(Recruitment in clinical trials)	Unchecked	21	6	15	22	33	22	
Module 3	Checked	11	3	5	26	24	38	0.18
(Why do clinical trials take so long? Trial enrollment process and barriers)	Unchecked	10	4	13	26	48	39	
Module 1	Checked	0	0	3	27	28	48	<0.001
(Barriers to research and historical events)	Unchecked	21	7	15	25	44	29	

Of the top 5 rated modules in Seminar 1, modules 2, 3, 5, 6, and 8 were statistically significant in providing a score of 3 or higher in understanding those modules. Of the top 5 rated modules in Seminar 2, modules 1, 4, and 5 were statistically significant in providing a score of 3 or higher in understanding those modules.

a Chi-square test was used or fisher exact test were used whenever appropriate. Fisher’s exact test was used when the cell count was under 5. Significant p values were marked red and defined as p < 0.05.

## Discussion

We found that the Community Health Worker Research Training, developed as an equitable partnership between academic researchers and CHWs, enhanced the research knowledge, awareness, and skills of CHWs as evidenced by their improvements in knowledge. CHWs often lack adequate and standardized training in the fundamentals of clinical research and understanding of the importance of increasing diversity and equity in research (George et al., 2021; Plasencia et al., 2023). This educational curriculum aims to address this gap and build CHWs’ capacity to serve as champions of clinical research within their communities, which can help promote and address the needs of community members. This training also addresses the gap in the research workforce by potentially expanding its capacity to incorporate CHWs onto research teams. It is crucial that the development of a curriculum build upon current CHW training competencies and take into account the past experiences of CHWs to improve organizational readiness and their seamless integration into healthcare systems (George et al., 2021). We accomplished this goal by leveraging the knowledge of CHWs and other key stakeholders to build upon the competencies and experiences of CHWs while incorporating research specific training. In recent years, there has been a leveling of the competency framework that includes fundamental, skilled, and advanced levels demonstrating increased competencies that occur through experience and career growth (Sonstein et al., 2020). Our CHW training provides a fundamental level of competency in clinical research with the following objectives: 1) Improve understanding of the foundations of clinical research, 2) Improve understanding of the importance of research diversity and equity in clinical research, and 3) Enhance research health literacy for CHWs through culturally appropriate trainings.

Over the course of developing and launching this training, we found that there is a pervasive need for CHWs in New York City and the U.S. broadly to expand their knowledge of clinical research. Murphy et al. identified a similar gap and developed an online course for CHWs on research best practices (Murphy et al., 2023). The training was received positively as both useful and relevant by both English-speaking and Spanish-speaking CHWs, further demonstrating a need for this training. Within the first 5 weeks of launching this training, we were able to engage 428 participants from 197 institutions across the country. Of the 247 survey respondents, one-third reported that it was their first time taking a research training and nearly all (99%) reported that this training provides practical tips and guidelines for CHWs that will be used in their day-to-day roles. These results demonstrate that the curriculum fulfilled an unmet need in CHW training. All respondents stated that they would recommend this training to others and, in fact, many stated that they learned about this training as a referral from a colleague or recommendation from a CHW organization. These findings provide evidence of feasibility, acceptability, and satisfaction which can inform larger-scale roll-out of the training. Although all respondents recommended this training to their colleagues, Seminar 1 was rated more highly than Seminar 2, suggesting that there is a greater need for foundational-level training in clinical research rather than understanding a more holistic and comprehensive research history. In reviewing survey write-in responses, many also provided suggestions for future training modules, including understanding research barriers in rural health and exploring the myths and misconceptions of research within underserved communities.

**FIGURE 5:**
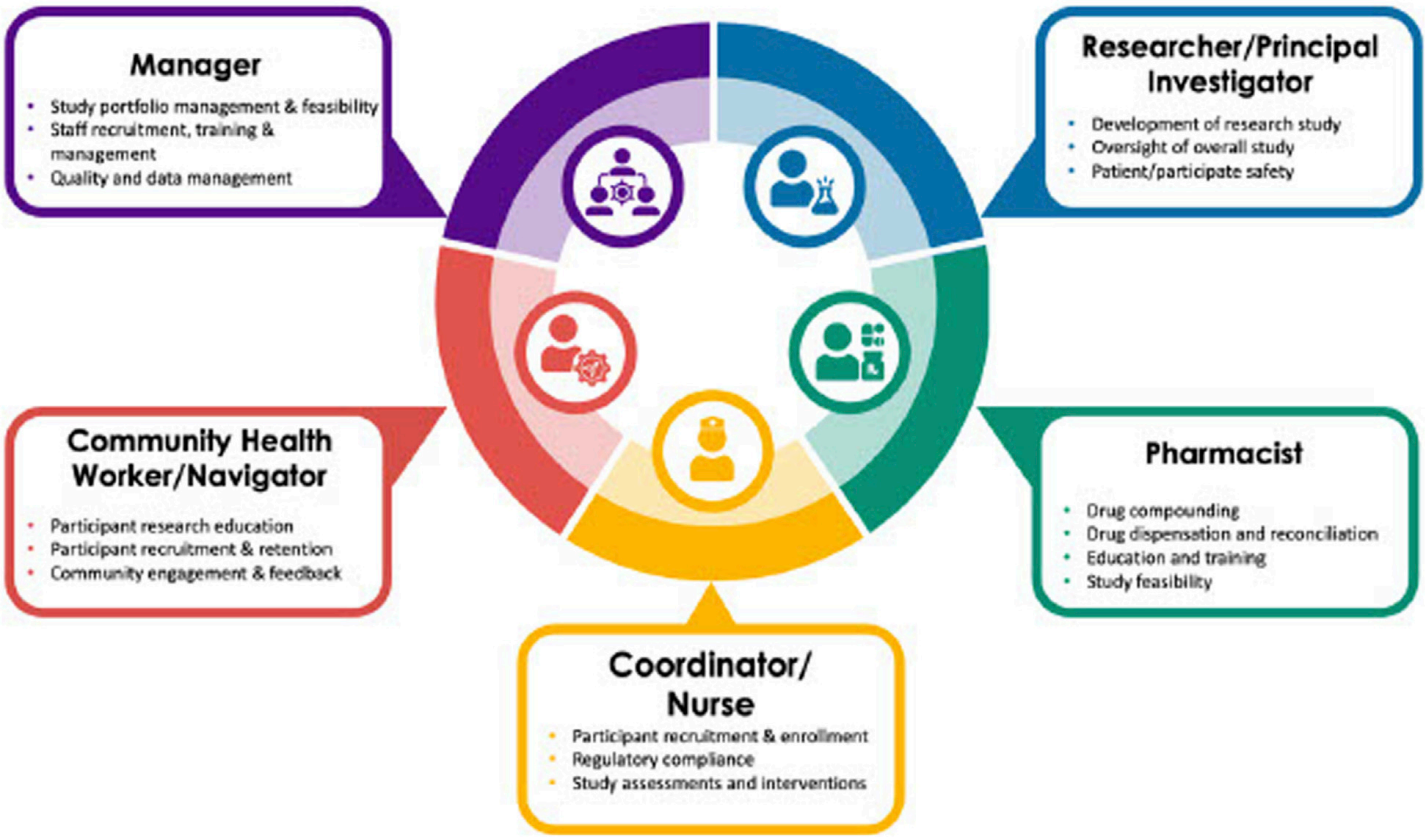
Community-Centered Integrated Research Team. Community-centered research teams integrated with principal investigators, pharmacists, managers, coordinators, nurses, and community health workers and research navigators. Creating such teams with CHWs and the community in mind will enable a bi-directional channel from the community to the research team and back, thus ensuring that the community and its needs are studied and that all patients are provided with equitable access to research opportunities.

In May 2023, the FDA released guidance on decentralized clinical trials, highlighting the importance of engaging the community and recruiting and retaining diverse populations (Silver Spring and MD: Center for Drug Evaluation and Research, 2023). As research protocols become more decentralized, teams will need to become more community-focused to reach historically underserved groups. This requires a rethinking of the research workforce and the framework for how research teams are developed. As Figure 5 demonstrates, future teams will need to be agile and dynamic in their composition. They will require the involvement of a variety of experts, including researchers, nurses, coordinators, pharmacists, and CHWs. Creating such teams with CHWs and the community in mind will enable a bi-directional channel from the community to the research team and back, thus ensuring that the community and its needs are studied and that all patients are provided with equitable access to research opportunities. Given their training to work within community settings and understand the needs of the communities they serve, CHWs will play an increasingly pivotal role in the research workforce in conducting clinical research and delivering interventions. Research-trained CHWs can lead in a variety of tasks, including navigating community members to studies open to enrollment, participating on community advisory boards for research, educating community members on the importance of research, and serving on academic research teams to assist with recruitment and delivery of community-based interventions. As members of the research team, CHWs can take on leadership roles and, with the input of stakeholders, help design interventions tailored to the unique needs, culture, and context of the populations they serve. Focus groups conducted by Killough et al. demonstrated that CHWs have a need for transparency and effective communication from researchers (Killough et al., 2023). To promote research engagement with diverse populations, the study suggests involving CHWs from the beginning of the research process, focusing on collaboration rather than persuading them of the value of research, addressing confidentiality concerns, and prioritizing dissemination of research findings in accessible ways (Killough et al., 2023). This training helps establish a research-ready CHW workforce that can be formally incorporated into research teams going forward.

There were several limitations of our training and its evaluation. One limitation is that the training provides only a fundamental level of knowledge related to the JTF core competencies. While it introduces CHWs to research concepts, this level of training may not be sufficient for CHWs to actively participate as members of the research team. Future trainings can build on this curriculum by incorporating competencies at the skilled and advanced levels, as defined by the Joint Task Force for Clinical Trial Competency (Sonstein and Jones, 2018). Further, this training was developed by academic researchers and CHWs at NYU Langone Health, located in New York City, and as such did not include specific modules geared toward rural populations as well as racial or ethnic minorities that do not reside in NYC, which may be important for CHWs in other parts of the country. Although we collected organizational affiliations in our post-exit survey, we did not collect demographic information of all trainees, making it difficult to assess generalizability of findings. It also did not include disease-specific research training required for recruitment of patients into specific disease-focused research protocols. This virtual, asynchronous training provides many advantages, including access, convenience, and a pace-based format, as well as the potential to add future modules depending on need. However, it lacks the ability to solicit real-time feedback and discussion through skill-based exercises and concept exploration that an in-person or synchronous training may offer. As we continue to disseminate the training to institutions nationwide, we will assess what other competencies may be needed and partner with other hubs in the Clinical & Translational Science Award (CTSA) network to build out additional training modules. Our evaluation also had limitations in that it only assessed knowledge and self-reported skill attainment. Although respondents were required to answer all quiz questions correctly to complete the modules, the FOCUS and RISE training platforms do not provide information on the number of times respondents re-took the quiz to achieve the passing score. Future studies will assess the impact of the training on changes to attitudes and behaviors. The training did not include a pre-survey to establish a baseline level of understanding of clinical research knowledge. We only used a retrospective survey to assess participant change in knowledge and perceptions of the training itself. We will incorporate pre-post test design into the training going forward in order to further evaluate knowledge uptake.

Our immediate plans for the future include dissemination of the training across the CTSA network to ensure that CHWs are trained within those hubs across the country. We will also engage specific academic institutions and community-based organizations (CBOs) which employ CHWs but may not be affiliated with the CTSA network. In addition, based on exit survey responses, we are also developing training materials that can be utilized by CHWs in the field as a reference for frequently asked questions they may encounter from community members when discussing the importance of clinical research. These documents will include links to studies that are open to enrollment, making it easy for CHWs to navigate patients to eligible research opportunities. We will also develop community-facing materials that include basic information about clinical research and opportunities for participation that can be distributed to clients who are engaged in conversations about research. These materials will be vetted by health literacy experts to ensure that all information is presented at an appropriate reading level and context for diverse populations. We also plan to translate these materials into different languages, which will be particularly valuable in cities with large multi-lingual immigrant populations.

As we develop the future research workforce, it is imperative to expose CHWs to different research approaches and promote community-based research (Killough et al., 2023). Schleiff et al. recommend that as research becomes more decentralized and community-focused, CHW training should include skill-based courses, clinical and public health courses, as well as certifications and degrees (Silver Spring and MD: Center for Drug Evaluation and Research, 2023). Further, Olaniran et al., suggest that future training frameworks should focus on competencies or educational qualifications (Olaniran et al., 2017). Clinical research should be formally identified as one of these competencies and incorporated into education and training curriculums for CHWs. Our long-term goals include expanding modules into different topics to meet the needs of diverse populations, incorporating competencies at the skilled and advanced levels, and including disease-specific trainings. In addition, we plan to work with Kingsborough Community College to incorporate the curriculum into their Community Health Worker Training Program, which is a free, credited didactic program carried out in collaboration with the NYU Family Health Centers, one of the largest federally qualified health center networks in the country. As part of our evaluation strategy, we will longitudinally track participant outcomes to understand whether CHWs who complete the training ultimately pursue roles in clinical research. As part of a larger recruitment effort, we are also exploring the possibility of educating CHWs on protocols that recruit large populations, rare-diseases, or hard-to-reach communities. Forming collaborative cross-disciplinary research teams can be challenging, as individuals with varied training and expertise in different fields must work together to integrate under a single research endeavor. We will take a team science approach when incorporating CHWs onto research teams to ensure that all members’ perspectives are considered and the linguistic, cultural, and technical expertise of CHWs are recognized and fully optimized. As we expand our training, we plan to integrate CHWs into the clinical research team through a step-wise approach. CHWs will first be trained to assist with translations/interpretations, patient navigation, reducing financial toxicities, and conducting community education on research protocols. By upskilling with additional trainings, CHWs will ultimately be able to serve in more advanced roles, such as performing clinical assessments, assisting with regulatory submissions, and collecting and entering data. This will provide career advancement and a more well-defined path into a career in research.

The future of the clinical research workforce relies on the strengthening of a community-based workforce of clinical research professionals (CRPs), as it is integral to increasing diversity, decentralizing research, and ensuring that underserved populations have access to research opportunities in advancing clinical research as a care option (CRAACO). Clinical research professionals (CRPs) are the bedrock of clinical research and are comprised of a variety of members of the clinical research workforce beyond the principal investigator (e.g., coordinators, data analysts, nurses, regulatory professionals, project managers) (Freel et al., 2023). CHWs are critical, versatile, and effective members of the healthcare workforce that advocate for communities, connect clients to resources, and improve the quality of care of patients (Landers and Levinson, 2016). Yet they are some of the lowest-paid healthcare professionals with lack of career advancement opportunities, resulting in turnover and attrition (Smithwick et al., 2023). A focus group study conducted by the Center for Community Health Alignment indicated that creating specialized training should be the main factor for CHW career advancement (Smithwick et al., 2023). Future endeavors should create direct pathways for further education, specialized professional development, and integration into clinical spaces and research, giving rise to a nationally trained CHW workforce that can help improve participation rates and be prepared for future pandemics (Lau et al., 2021; Ahmed et al., 2022; Klein et al., 2022; Rodriguez, 2022; Smithwick et al., 2023). CHWs are primed to take on these roles as they are healthcare professionals with the necessary skills in recruitment, patient intervention, data collection, education, and health promotion.

The clinical research profession is in crisis with high staff turnover, lack of quality training, and high barriers to entry that require 2 years of research experience (Stabile et al., 2023). For every person seeking a position in clinical research, there are seven jobs posted with job growth expected at a rate of 9.9% by 2026 (Freel et al., 2023). Furthermore, there is a dearth of diverse, patient-facing healthcare research professionals, which exacerbates efforts to recruit diverse patient populations to research studies. Freel et al., outline key areas for workforce development and regeneration, including clear identity and visibility of CRPs, baseline standards for training and jobs roles, raising awareness, universal competency-based assessments, and increasing diversity in the CRP workforce (Freel et al., 2023). We are attempting to address these imperatives for clinical research workforce regeneration through the CHW research training and workforce development by 1) promoting the profession to other lateral members of the healthcare system, 2) establishing a standard for career development for CHWs entering research roles, 3) raising awareness about CHWs in the research workforce, 4) elevating the standards of research CHWs through module-based training, 5) expanding and defining the research roles for CHWs, and 6) diversifying the workforce by attracting individuals who work within the communities they serve. As we continue to expand our trainings and tools, we hope to reduce the barriers to entry for CHWs to be formally incorporated into the research team. The future of the clinical research workforce relies on research-trained CHWs, as they are integral to our mission of increasing the diversity of research professionals, decentralizing research, and ensuring that underserved populations have access.

The project described was supported by the National Center for Advancing Translational Sciences (NCATS), National Institutes of Health, through Grant Award Number UL1TR001445, NYU Community Service Plan, Perlmutter Cancer Center, and the Beatrice W. Welters Breast Health Outreach & Navigation Program. The content is solely the responsibility of the authors and does not necessarily represent the official views of the NIH. Nadia Islam’s time was partially supported by Centers for Disease Control and Prevention (CDC) grant number U48DP006396 and NIH National Heart, Lung, and Blood Institute (NHLBI) CEAL nonfederal 1OT2HL156812-01, Westat sub-OTA no: OT2HL158287.

## Data Availability

The raw data supporting the conclusion of this article will be made available by the authors, without undue reservation.
